# Unveiling the genetic tapestry of Kohistan: a population genetic analysis of autosomal STRs in a South Asian population

**DOI:** 10.1186/s12864-026-12641-x

**Published:** 2026-02-13

**Authors:** Atif Adnan, Allah Rakha, Hao Dong Chen, Muhammad Ilyas, Muhammad Farhat Ullah, Shahid Nazir, Chuan Chao Wang, Hongbo Wang

**Affiliations:** 1 School of Medical Science, Shandong Xiehe University, Jinan, Shandong Province 250109 China; 2 Department of Forensic Sciences, University of Health Sciences, Lahore, Punjab 54600 Pakistan; 3 Department of Anthropology and Ethnology, Institute of Anthropology, School of Sociology and Anthropology, Xiamen University, Xiamen, Fujian Province 361000 China; 4 Center for OMIC Sciences, Islamia Collage University Peshawar, Peshawar, KPK Pakistan; 5 National Centre for Bioinformatics, Quaid-I-Azam University, Islamabad, 44000 Pakistan; 6 Ministry of Education Key Laboratory of Contemporary Anthropology, Department of Anthropology and Human Genetics, School of Life Sciences, Fudan University, Shanghai, 200438 China; 7 State Key Laboratory of Genetic Engineering, Center for Evolutionary Biology, School of Life Sciences, Fudan University, Shanghai, 200438 China; 8 Department of Human Anatomy, School of Basic Medical Sciences, Shenyang Medical College, Shenyang, Liaoning Province 110034 China; 9 Liaoning Province Key Laboratory for Phenomics of Human Ethnic Specificity and Critical Illness, Shenyang Medical College, Shenyang, Liaoning Province China

**Keywords:** Kohistani population, Forensic genetics, Population history, Autosomal STRs, South asia

## Abstract

The Kohistani population of northern Pakistan, an understudied group with distinct geographic, ethnic, and cultural characteristics, presents a unique opportunity to explore genetic diversity in South Asia. We analyzed 21 autosomal STR loci in 200 unrelated Kohistani males using the GlobalFiler™ PCR Amplification Kit, identifying 329 distinct allele combinations with allele frequencies ranging from 0.0025 to 0.2975. Forensic parameters revealed high informativeness, with a combined power of discrimination (CPD) of 0.999999999999999999999999999999378 and a combined power of exclusion (CPE) of 0.9999336, though loci like TPOX showed reduced diversity (GD: 0.7389, Ho: 0.545) compared to Pashtuns (GD: 0.73–0.92) and Punjabis (GD: 0.75–0.95), reflecting endogamy. After sequential Bonferroni correction, no significant deviations from Hardy-Weinberg equilibrium were detected, but initial deviations and linkage disequilibrium in 16 locus pairs suggest genetic drift, similar to patterns in Baloch populations. Analyses of genetic distances, multi-dimensional scaling (MDS), principal component analysis (PCA), STRUCTURE, and phylogenetic trees showed a genetic affinity between the Kohistani population and neighboring South Asian populations. This genetic connection aligns with linguistic, ethnic, and geographical classifications, placing Kohistani in close proximity to populations like Punjabi, Pashtuns, and Bangladeshi, reflecting shared ancestry and regional gene flow. These findings highlight Kohistani’s genetic structure, shaped by isolation and endogamy, and contribute to understanding South Asian genetic diversity.

**Supplementary Information:**

The online version contains supplementary material available at 10.1186/s12864-026-12641-x.

## Introduction

**Fig. 1:**
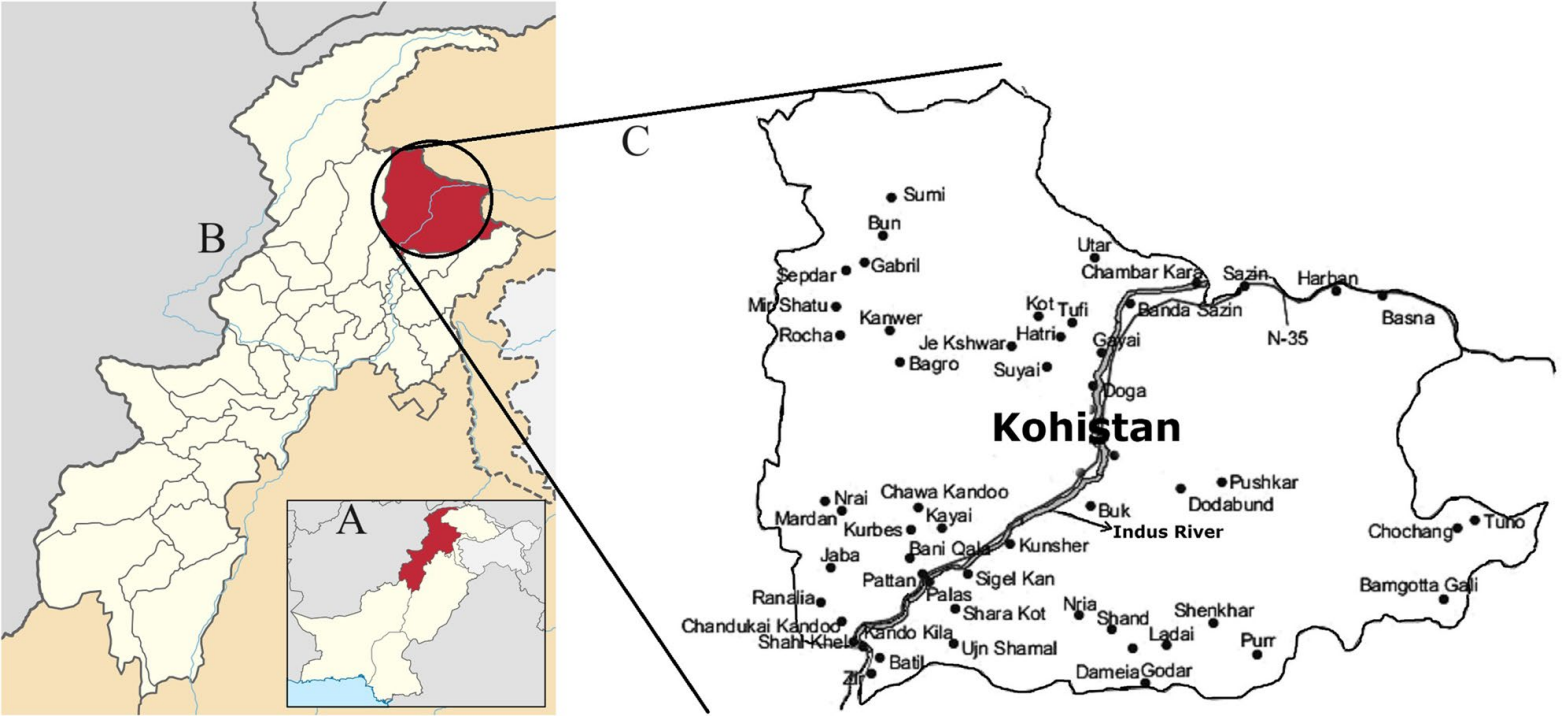
Location of the sampling collection area on map

Northern Pakistan, characterized by its distinctive topography of high mountains and deep river gorges, along with its diverse ethnicities and languages, is an area of significant research interest due to its rich cultural heritage and ethnic diversity. This region serves as a crucial connection point between the Himalayas, the Hindukush, and the Karakoram Mountain ranges. Nestled within this rugged landscape is Kohistan, a hilly region geographically positioned in the northernmost part of Khyber Pakhtunkhwa (KPK) province, between Pakistan’s western and eastern borders. The Indus River divides Kohistan into two parts: Hazara and Swat, which were unified in 1976 to form the Kohistan district (Fig. 1), encompassing an area of 7,492 square kilometers. This rugged terrain has fostered the development of distinct ethnic groups and languages, isolated yet interconnected through centuries of migration and trade. Kohistan is home to various ethnic groups, the most prominent being the Kohistani people, who speak the Kohistani language. Classified as a Dardic language belonging to the Indo-European family [1], Kohistani plays a pivotal role in shaping community identity [2]. However, among the Dardic languages, Kohistani remains the least studied [3]. The origins and genealogy of the Kohistani people are largely unknown due to a scarcity of historical records and established traditions. Ancient mentions of the region appear in the Rig Veda, as well as in Greek and Chinese literature, reflecting its historical significance as a Silk Road crossroads. Throughout history, Buddhist, Hindu, and Muslim cultures have influenced Kohistan [4, 5], leaving an indelible mark on modern Kohistani society, which continues to reflect a blend of these traditions. This cultural diversity aligns with the complex genetic history of South Asia, shaped by ancient migrations and admixture events, as evidenced by genomic studies of the region [6, 7]. Despite its cultural richness, Kohistan has the lowest literacy rates in Pakistan, with 17% for males and 3% for females, and a population of 784,711 with an average annual growth rate of 2.70% (https://www.pbs.gov.pk/).

The Kohistani population, residing in a geographically isolated region of northern Pakistan, represents an understudied ethnic group with unique linguistic and cultural characteristics. Centuries of migration along the Silk Road have contributed to Kohistan’s rich tapestry of ethnicities, making it a unique case study for exploring human genetic diversity. Social practices such as endogamy, prevalent within Kohistani society, likely influence the population’s genetic structure, contributing to patterns of genetic drift and reduced diversity. Despite its historical significance as a crossroads between South and Central Asia, the genetic composition and population history of the Kohistani people remain poorly understood due to limited genetic studies.

To address these gaps, this study investigates the genetic structure, ancestry, and relationships of the Kohistani population using autosomal Short Tandem Repeat (STR) markers. STRs, known for their high polymorphism and forensic utility, enable robust comparisons with global datasets such as the 1000 Genomes Project [8], which provides a comprehensive reference for human genetic variation across 26 worldwide populations. These markers are DNA sequences with repeating patterns of 2 to 7 base pairs found throughout the human genome, characterized by stable polymorphisms, short sequence lengths, and even chromosomal distribution, facilitating easy identification and analysis using PCR and sequencing [9, 10]. Due to their highly polymorphic and informative nature, STRs are considered the gold standard in forensic investigations, including paternity testing, sexual assault cases, kinship analysis, and missing person identification [11–15]. Their widespread use in forensic genetics is well-documented in foundational studies that established STRs as reliable markers for human identification and kinship analysis [14, 15]. In this study, we genotyped 200 unrelated individuals from the Kohistani ethnic group of Pakistan using a 6-dye GlobalFiler PCR Amplification kit (Thermo Fisher Scientific, Inc., Waltham, MA, USA). This kit includes 21 commonly used autosomal STR loci (D8S1179, D21S11, D7S820, CSF1PO, D3S1358, TH01, D13S317, D16S539, D2S1338, D19S433, VWA, TPOX, D18S51, D5S818, FGA, D12S391, D1S1656, D2S441, D10S1248, D22S1045, and SE33), along with three gender determination loci (Amelogenin, Yindel, and DYS391). By focusing on unrelated male individuals, we aim to minimize bias from familial relatedness while capturing the population’s genetic diversity. This study employs an ethnogenetic approach, integrating genetic, linguistic, and geographical data to provide a comprehensive understanding of the Kohistani population’s origins and evolutionary history. Such insights not only enrich our knowledge of human diversity but also contribute to forensic science and the preservation of cultural heritage in this historically significant region.

## Materials and methods

### Study population and DNA extraction

This study aimed to characterize the genetic structure of the Kohistani population using 21 autosomal short tandem repeat (STR) loci amplified with the GlobalFiler™ PCR Amplification Kit. Autosomal STRs were selected for their high discriminatory power, compatibility with international forensic and population databases, and proven utility in elucidating genetic diversity attributes established since their foundational use in human identification by Edwards et al. (1991) [16]. A total of 200 unrelated males were recruited from Upper and Lower Kohistan, districts within Khyber Pakhtunkhwa (KPK), Pakistan, geographically divided by the Indus River (Fig. 1). To ensure ethnic homogeneity, participants were required to self-identify as Kohistani, confirm through a detailed questionnaire that both parents were of Kohistani descent, and demonstrate fluency in the Kohistani language, a key cultural identifier. Residency in either Upper or Lower Kohistan was verified with the assistance of local collaborators, including community elders. Individuals with known recent admixture (e.g., one non-Kohistani parent) were excluded. The exclusive inclusion of males was necessitated by logistical and cultural constraints in this remote region, where social norms and ethical considerations limited access to female participants. The sample size of 200 was determined based on statistical power calculations recommended for STR-based population studies [17, 18], offering a practical balance between representativeness and feasibility given Kohistan’s estimated population of approximately 784,711. Selecting unrelated individuals minimized bias from familial clustering, and the cohort size aligns with those used in comparable studies of isolated populations [10, 11].

Sample collection was coordinated by the Research Registry of the Office of Research, Innovation, and Commercialization (ORIC) at Islamia College University, Peshawar, and carried out by a trained phlebotomy team to ensure standardized protocols and participant safety. De-identified samples, labeled only with sex and ethnic origin, were subsequently transferred to the research team to preserve confidentiality. The study was approved by the Bioethics Review Committee of Islamia College University (Approval No.: 866/ORIC/ICP, 23 April 2023) and conducted in accordance with the Declaration of Helsinki (World Medical Association, 2013) and Pakistani national regulations. Written informed consent was obtained from all participants. Genomic DNA was extracted from blood samples using the ReliaPrep™ Blood gDNA Miniprep System (Promega, Madison, USA) following the manufacturer’s protocol. DNA sample concentrations were measured using a NanoDrop spectrophotometer (Thermo Scientific, Wilmington DE, USA). The final DNA concentration was adjusted to 1–2 ng/µl.

###  PCR amplification, and genotyping

The extracted DNA samples were subjected to PCR amplification targeting 24 genetic markers, including 21 autosomal STR loci and three gender determination loci (Amelogenin, Yindel, and DYS391). Amplification was performed using the commercial kit on a GeneAmp PCR System 9700 (Thermo Fisher Scientific company), following the manufacturer’s instructions. The resulting PCR products were analyzed on an 8-capillary ABI 3500 DNA Genetic Analyzer with POP-4™ polymer (Life Technologies). Genotypes were assigned using GeneMapper Software version 1.5 (Life Technologies). Allele nomenclature followed the allelic ladder provided with the kit [19], adhering to the guidelines of the International Society for Forensic Genetics (ISFG) [20, 21].

### Quality control

Negative (autoclaved deionized H₂O) and positive (AmpFlSTR^®^ Control DNA 9947 A) controls were included during DNA extraction, quantification, PCR amplification, and capillary electrophoresis. No amplification products were observed in the negative controls, while positive controls yielded genotypes consistent with known profiles.

### Data analysis

Allele frequencies and key forensic statistical parameters (e.g., gene diversity, polymorphic information content, power of discrimination, and power of exclusion) were calculated using STRAF [17]. Additionally, principal component analysis (PCA) was performed using STRAF to visualize the genetic structure and relationships of the Kohistani population with other populations. Hardy-Weinberg equilibrium (HWE), linkage disequilibrium (LD), and observed heterozygosity (Ho) were estimated using Arlequin 3.5 [22]. Autosomal STR data from the 1000 Genomes Project dataset were extracted using HipSTR (Haplotype inference and phasing for Short Tandem Repeats) [23]. Autosomal STR genotype data for 26 worldwide populations from the 1000 Genomes Project Phase 3, previously generated using HipSTR [17], were obtained from publicly available datasets [24]. These data were used for comparative analyses, including pairwise Fst genetic distances, multi-dimensional scaling (MDS), principal coordinates analysis (PCoA), and STRUCTURE, to assess the genetic relationships of the Kohistani population with global populations. Pairwise Fst genetic distances between the Kohistani population and 26 other global populations from the 1000 Genomes Project, representing five continents [8], were calculated using Arlequin 3.5 [22]. Nei’s genetic distances between the Kohistani population and 22 Asian populations along with 26 populations from 1000 genome project were assessed using the Phylip 3.695 program [25]. The Phylogenetic tree [26] was visualized using Mega7 software [27]. Multi-Dimensional Scaling (MDS) and Principal Coordinates Analysis (PCoA) were used to visualize genetic relationships among populations based on Nei’s genetic distances. MDS is a statistical method that projects pairwise genetic distances into a low-dimensional space (typically two dimensions) to minimize distortion of the original distances, producing two-dimensional plots to illustrate genetic affinities. PCoA, a specific form of metric MDS, applies eigenvalue decomposition to the distance matrix to maximize the variance explained by principal coordinates, providing a complementary visualization that quantifies the proportion of variance captured by each axis. In this study, MDS was used to compare the Kohistani population with global (1000 Genomes Project) and regional (Asian and South Asian) populations, while PCoA was also applied to regional populations to confirm clustering patterns, with PC1 and PC2. Both analyses were performed using Nei’s genetic distances calculated in Phylip 3.695 [19], with visualizations generated in the R statistical environment. Ancestry component deconstruction was investigated using STRUCTURE v.2.3.4 [28]. The model-based analysis employed a burn-in period of 100,000 iterations and a Markov Chain Monte Carlo (MCMC) step of 100,000 iterations, utilizing the ‘independent allele frequencies’ and ‘LOCPRIOR’ models with K values ranging from 2 to 15.

## Results and discussion

### Forensic parameters

We genotyped 200 unrelated Kohistani males using the 21 autosomal STR loci (D8S1179, D21S11, D7S820, CSF1PO, D3S1358, TH01, D13S317, D16S539, D2S1338, D19S433, VWA, TPOX, D18S51, D5S818, FGA, D12S391, D1S1656, D2S441, D10S1248, D22S1045, and SE33), along with three gender determination loci (Amelogenin, Yindel, and DYS391). Allele frequencies are provided in Supplementary Table 1. A total of 329 distinct allele combinations were observed, with frequencies ranging from 0.0025 to 0.2975. SE-33 was the most polymorphic locus (50 allele combinations), while CSF1PO and D16S539 were the least (9 combinations each). Gene diversity (GD) ranged from 0.7389 (TPOX) to 0.9639 (SE-33), and polymorphic information content (PIC) ranged from 0.7 (TPOX) to 0.9601 (SE-33) and power of discrimination (PD), calculated as 1 - PM, ranged from 0.8852 (TPOX) to 0.98995 (SE-33). The combined PD (CPD) was 0.999999999999999999999999999999378, and the combined power of exclusion (CPE) was 0.9999336. Observed heterozygosity (Ho) ranged from 0.545 (TPOX) to 1 (SE-33), and power of exclusion (PE) ranged from 0.2299 (TPOX) to 1 (SE-33). The low GD (0.7389) and Ho (0.545) at TPOX indicate reduced diversity, likely due to endogamy and isolation (Table 1). Compared to Pashtuns from Khyber Pakhtunkhwa (GD: 0.73–0.92, Ho: 0.55–0.85 for TPOX) [29], Punjabis from Lahore (GD: 0.75–0.95, Ho: 0.6–0.9) [30], and Baloch (GD: 0.74–0.90, Ho: 0.58–0.82) [31], the Kohistani population shows greater homozygosity, reflecting a smaller effective population size and stricter endogamy. Bangladeshis [32], with a combined PD of 0.99991 for four STR loci including TPOX, exhibit higher diversity, likely due to less isolation. These forensic parameters demonstrate the high informativeness of most loci, underscoring the kit’s utility for individual differentiation and paternity testing within the Kohistani population.

**Table 1:** Forensic efficiency and statistical parameters on 21 autosomal STR loci in the Kohistani population from KPK, Pakistan (*n* = 200)

locus	Nall	GD	PIC	PM	PD	Hobs	PE	TPI
CSF1PO	9	0.799825	0.767996	0.0731	0.9269	0.645	0.348391	1.41
D10S1248	12	0.832794	0.808788	0.06755	0.93245	0.9	0.79542	5
D12S391	12	0.865013	0.847748	0.0474	0.9526	0.955	0.908498	11.11
D13S317	10	0.836165	0.812269	0.0607	0.9393	0.865	0.724634	3.7
D16S539	9	0.817682	0.791777	0.06495	0.93505	0.69	0.412961	1.61
D18S51	15	0.861266	0.843998	0.06025	0.93975	0.92	0.836433	6.25
D19S433	26	0.896917	0.886067	0.033	0.967	0.94	0.87762	8.33
D1S1656	13	0.87589	0.860322	0.04085	0.95915	0.93	0.857017	7.14
D21S11	21	0.887055	0.874022	0.0313	0.9687	0.73	0.476181	1.85
D22S1045	14	0.845639	0.826434	0.0695	0.9305	0.995	0.989976	99.5
D2S1338	14	0.889223	0.876162	0.03715	0.96285	0.925	0.846721	6.67
D2S441	12	0.822757	0.796831	0.07465	0.92535	0.875	0.74469	4
D3S1358	12	0.797694	0.765779	0.0724	0.9276	0.66	0.369131	1.47
D5S818	12	0.813133	0.787533	0.08225	0.91775	0.965	0.929023	14.29
D7S820	10	0.836353	0.812915	0.061	0.939	0.885	0.764891	4.35
D8S1179	16	0.876429	0.861191	0.039	0.961	0.575	0.261948	1.18
FGA	26	0.910664	0.901161	0.02585	0.97415	0.99	0.979906	49.5
SE33	50	0.96386	0.960074	0.01005	0.98995	1	1	100
TH01	10	0.831078	0.80685	0.06575	0.93425	0.81	0.61773	2.63
TPOX	12	0.738897	0.700004	0.1148	0.8852	0.545	0.229999	1.1
vWA	14	0.837456	0.815557	0.0495	0.9505	0.74	0.492814	1.92

*Nall* Number of allels, *GD* gene diversity, *PIC* polymorphism information content, *Hobs*observed heterozygosity, *PD * power of discrimination, *PM*matching probability,*PE*power of exclusion, *TPI * typical paternity index

### Hardy-Weinberg equilibrium (HWE)

None of the 21 loci were initially in HWE, with an excess of homozygotes at TPOX (*p* < 0.05). After sequential Bonferroni correction [33] (adjusted p-value = 0.05 / 21 ≈ 0.00238), all loci conformed to HWE (Supplementary Table 2). The pre-correction deviations, particularly at TPOX, suggest endogamy or population substructure, common in isolated groups. Similar patterns occur in Baloch populations, where HWE deviations at TPOX persist pre-correction due to consanguinity. Pashtuns show fewer deviations, with most loci in HWE post-correction, indicating less inbreeding than Kohistanis. Punjabis typically conform to HWE without correction, reflecting random mating in larger populations. Indian populations [34] with endogamous communities also show HWE deviations, aligning with Kohistani patterns. The Kohistani population’s strong deviations underscore the impact of geographic isolation and cultural practices limiting gene flow.

### Linkage equilibrium (LE)

Exact tests revealed significant linkage disequilibrium (LD) in 80 pairwise locus comparisons (*p* < 0.05). After Bonferroni correction [27] (adjusted *p*-value = 0.05 / 210 ≈ 0.000238), 16 pairs remained in LD (D18S51/TPOX, D1S1656/D13S317, D1S1656/D8S1179, FGA/D8S1179, D1S1656/FGA, FGA/D2S441, D13S317/TPOX, D10S1248/D19S433, D2S441/vWA, D1S1656/D21S11, D8S1179/vWA, D21S11/vWA, FGA/D21S11, D2S441/D8S1179, D2S441/D21S11 and D21S11/D8S1179) (Supplementary Table 3). As many of these loci are located on different chromosomes, these significant results are unlikely to reflect true linkage disequilibrium due to physical linkage. Instead, they likely arise from demographic and cultural factors, such as genetic drift, endogamy, and a small effective population size, which are prevalent in the isolated Kohistani population. This is consistent with the observed excess of homozygotes at loci like TPOX (Sect. 3, Hardy-Weinberg Equilibrium) and aligns with patterns seen in other endogamous South Asian populations, such as the Baloch, where significant LD across unlinked loci has been attributed to population substructure and inbreeding [25]. Compared to Pashtuns, who show fewer significant pairs (e.g., 10 pre-correction), the Kohistani population’s higher number of significant associations reflects stronger genetic drift due to its smaller population size and stricter endogamy. Punjabis, with minimal significant LD, indicate random mating in a larger, less isolated population. These findings highlight the impact of Kohistani’s geographic isolation and cultural practices, such as endogamy, on its genetic structure, mimicking LD patterns typically associated with linked loci [28, 29, 35].

### Kohistani population ancestry content analysis with STRUCTURE

**Fig. 2:**
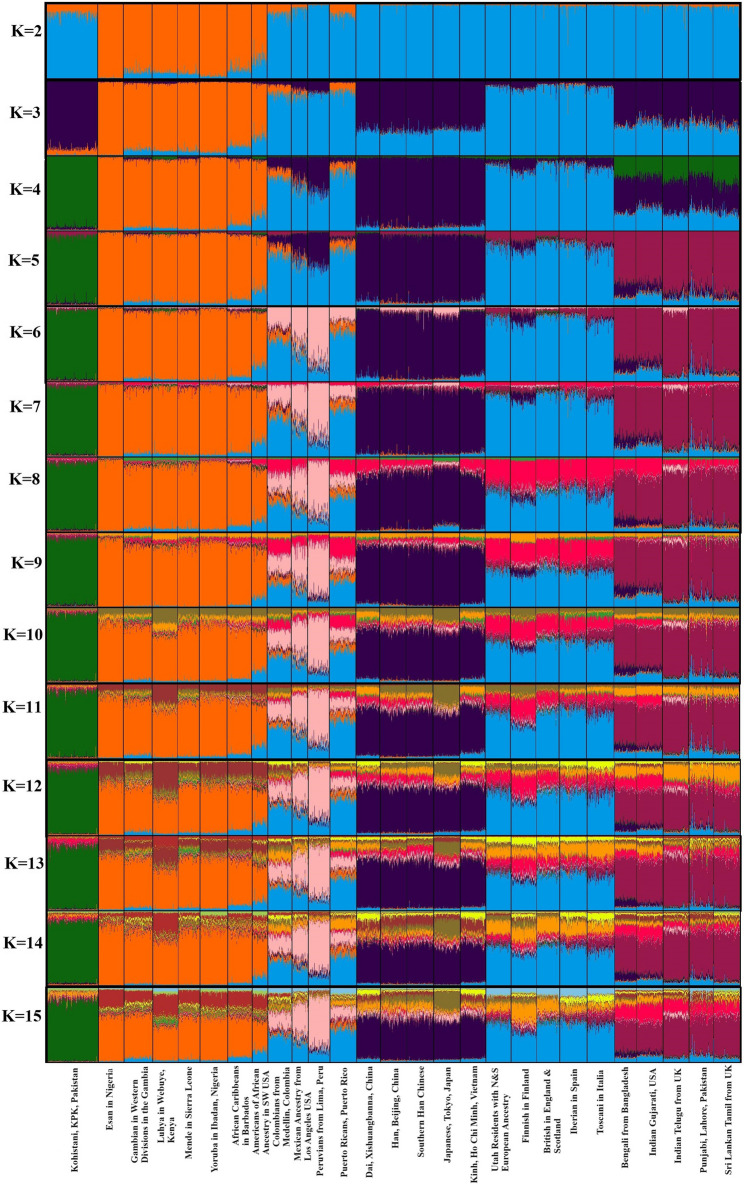
Structure analysis results between Kohistani population and 1000 genome populations (K = 2 to K = 15)

STRUCTURE analysis, incorporating data from the 1000 Genomes Project, provided insights into the ancestral composition of the Kohistani population alongside 26 global populations. The optimal number of ancestral populations was determined to be two (K = 2), with a high mean similarity score of 0.985 (Fig. 2). At this level, two primary ancestry components (blue and orange) were identified. The Kohistani population showed a predominance of the blue component (95%), slightly less than in neighboring South Asian populations (99%), suggesting a strong genetic affinity within South Asia while also indicating a distinct genetic profile for the Kohistani. Further analysis at K = 4 (mean similarity score 0.972) revealed four ancestry components (green, blue, purple, and orange), with the Kohistani population almost exclusively characterized by the green component (99.5%), a trait shared only with other South Asian populations. This distinct genetic signature highlights the unique genetic landscape of the Kohistani people, potentially reflecting historical migrations, genetic drift, or founder effects. These findings contribute to understanding genetic diversity within South Asia and emphasize the importance of studying underrepresented populations. The specific genetic components dominant in the Kohistani population suggest avenues for further research into the historical and evolutionary dynamics shaping South Asian genetic structure.

### Kohistani and 1000 genome population comparison

**Fig. 3:**
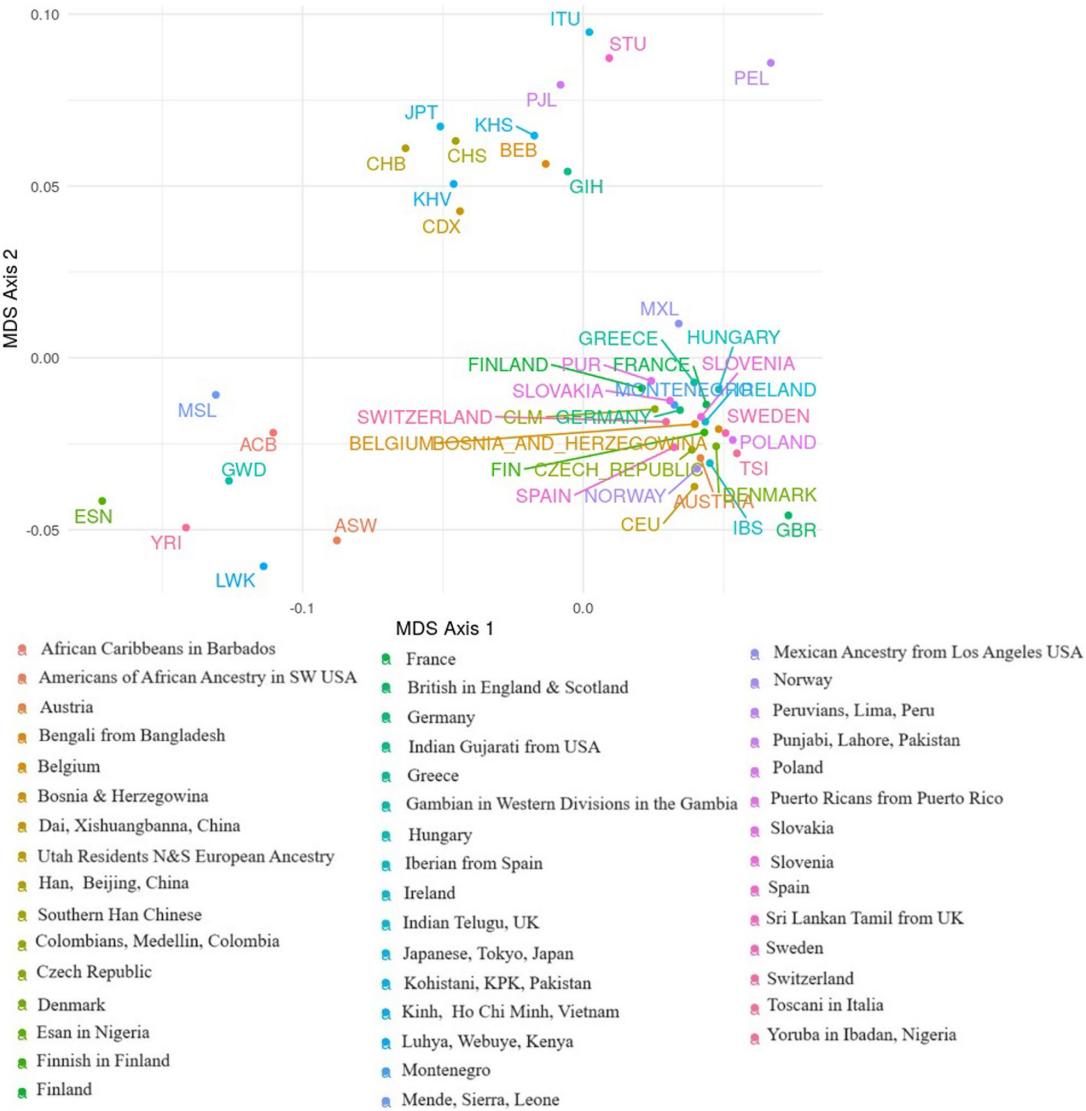
Two-dimensional plot from multi-dimensional scaling analysis of Nei’s values based on 21 autosomal STRs between Kohistani population, 1000 genome populations and European populations from STRidER

**Fig. 4:**
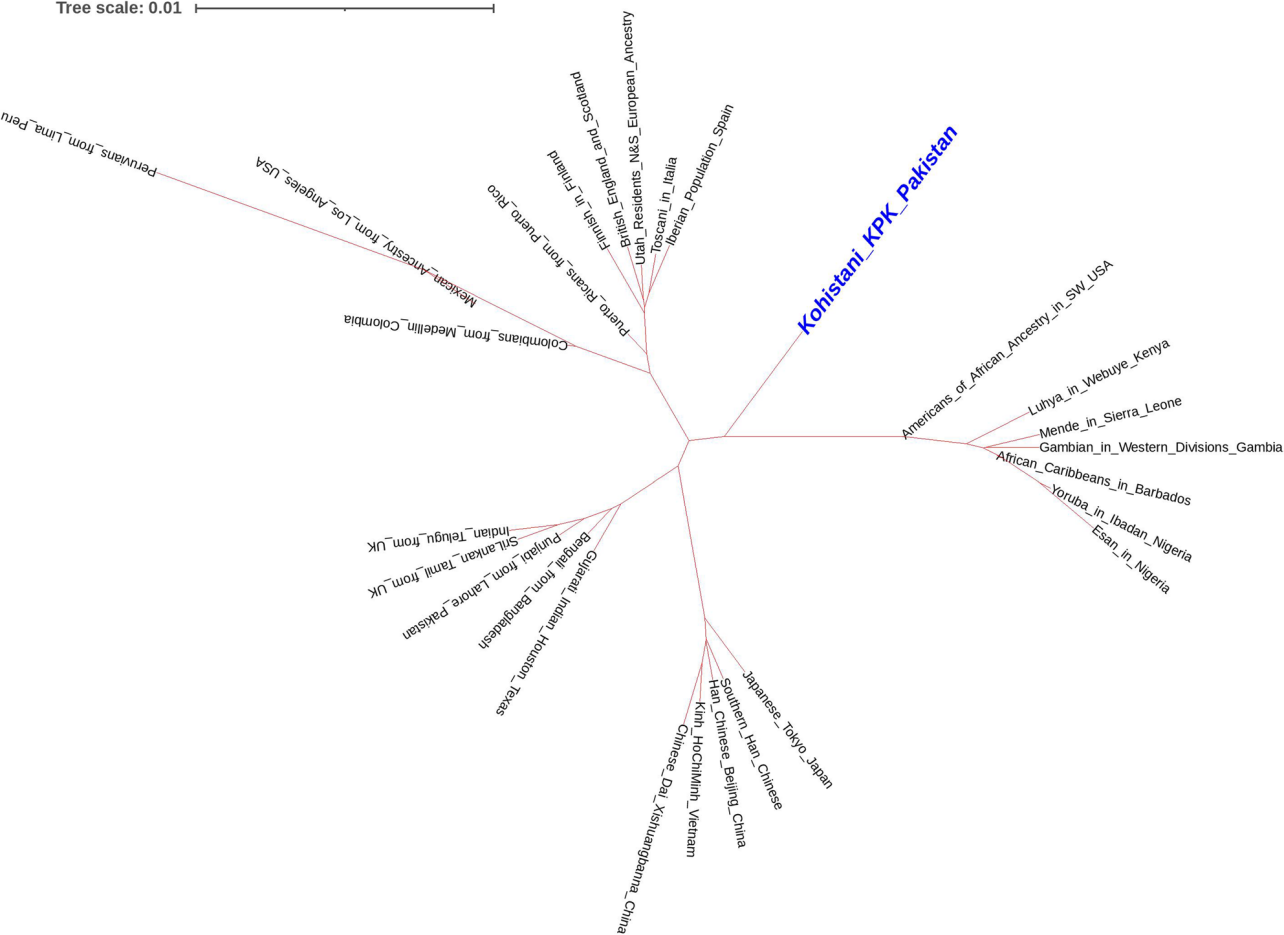
NJ Tree between Kohistani population and 1000 genome populations based on Nei’s formula

**Fig. 5:**
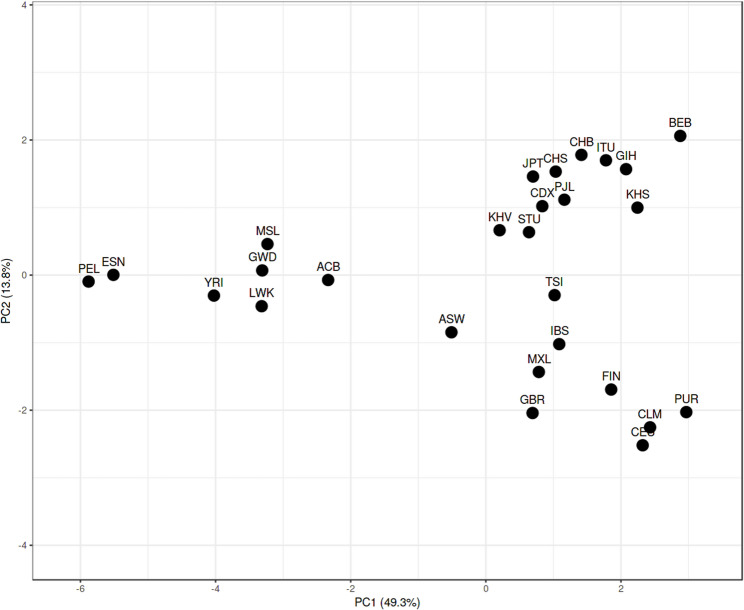
Principal component analysis (PCA) between Kohistani population and 1000 genome populations

Analysis of Fst genetic distances between the Kohistani population and 26 worldwide populations from the 1000 Genomes Project revealed the closest association with the Bangladeshi population in Bangladesh (0.0085), followed by the Punjabi population from Lahore, Pakistan (0.0094). The most distant associations were with Peruvians from Lima, Peru (0.0233) and the Esan population from Nigeria (0.0178) (Supplementary Table 4). A multi-dimensional scaling (MDS) plot based on Nei’s genetic distances placed the Kohistani population alongside other South Asian populations, including Punjabi from Lahore, Indian Gujarati from the USA, and Bengali from Bangladesh, indicating close genetic relationships (Fig. 3). The Kohistani population appeared genetically distinct from European and African population clusters, suggesting relative genetic isolation. A neighbor-joining tree, based on Fst genetic distances, similarly positioned the Kohistani population as genetically distinct yet closely related to other South Asian populations (Fig. 4). Principal component analysis (PCA), with PC1 explaining 49.3% and PC2 explaining 13.8% of the variance (Fig. 5), also showed the Kohistani population clustering with South Asian populations, differentiating them from African, East Asian, and, to a lesser extent, European groups, highlighting regional genetic homogeneity. The interactivity test, based on allele frequency profiles of the 21 autosomal STR loci, was conducted to assess genetic interactions between the Kohistani population and the 26 worldwide populations from the 1000 Genomes Project (Supplementary Fig. 1). The test revealed significant genetic interactions (*p* < 0.05 after Bonferroni correction) between the Kohistani population and South Asian populations, particularly the Bangladeshi (*p* = 0.0012) and Punjabi from Lahore (*p* = 0.0028), indicating strong genetic affinity consistent with shared ancestry and regional gene flow. In contrast, weaker interactions were observed with European (e.g., CEU, *p* = 0.124), African (e.g., Esan, *p* = 0.098), East Asian (e.g., CHB, *p* = 0.142), and South American (e.g., Peruvian, *p* = 0.115) populations, reflecting greater genetic differentiation due to geographic and historical barriers to gene flow. These results, visualized as a heatmap in Supplementary Fig. 1, complement the Fst genetic distances, MDS, and PCoA analyses (Figs. 3, 4 and 5), reinforcing the Kohistani population’s close clustering with South Asian populations and its distinctiveness from other global groups. The strong interactions with South Asian populations align with linguistic and geographic ties, such as the Dardic language heritage and proximity to neighboring groups like Pashtuns and Punjabis.

### Kohistani population comparison with Asian & South Asian populations

**Fig. 6:**
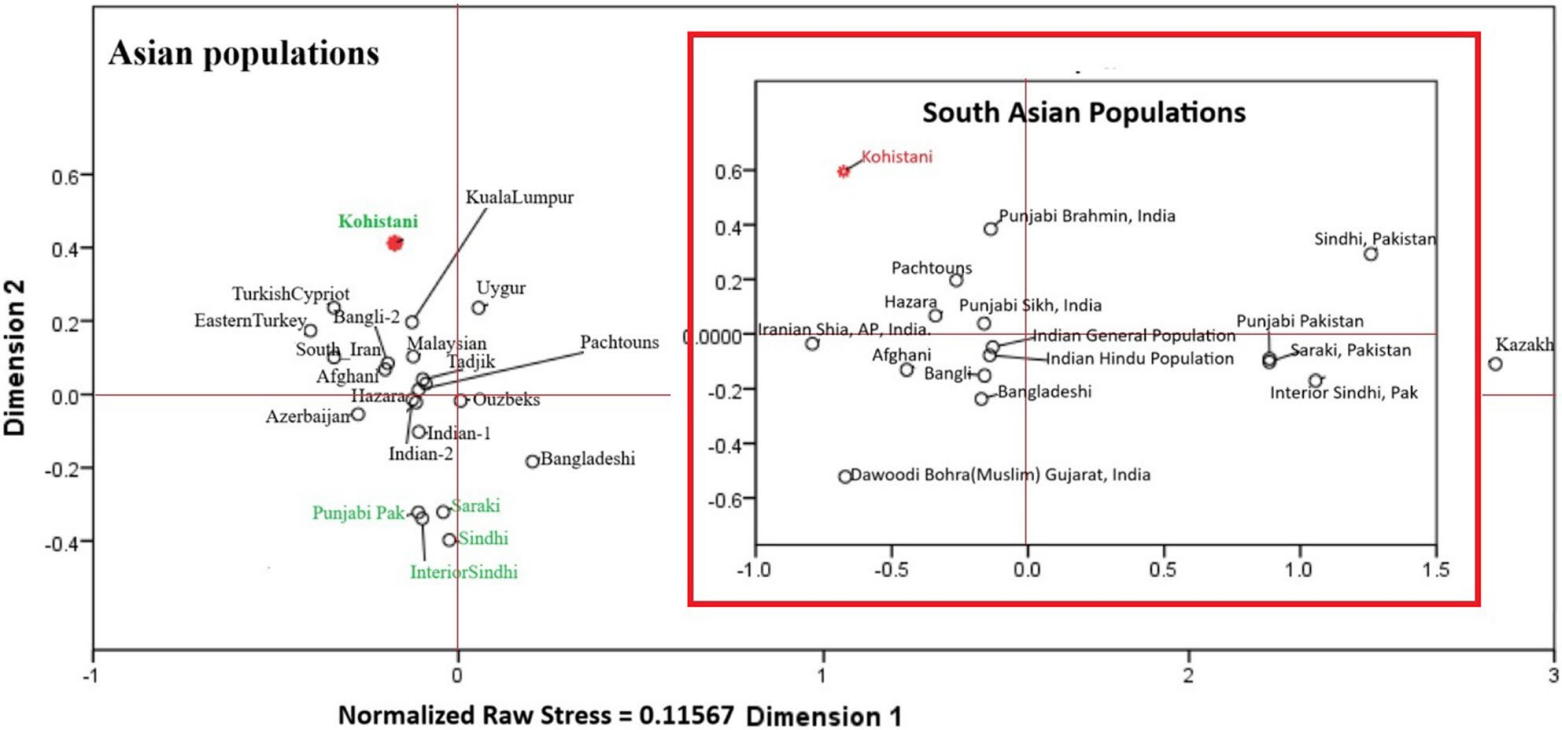
Two-dimensional plot from multi-dimensional scaling analysis of Nei’s values based on 15 overlapping autosomal STRs between Kohistani population, and 22 reginal populations

**Fig. 7:**
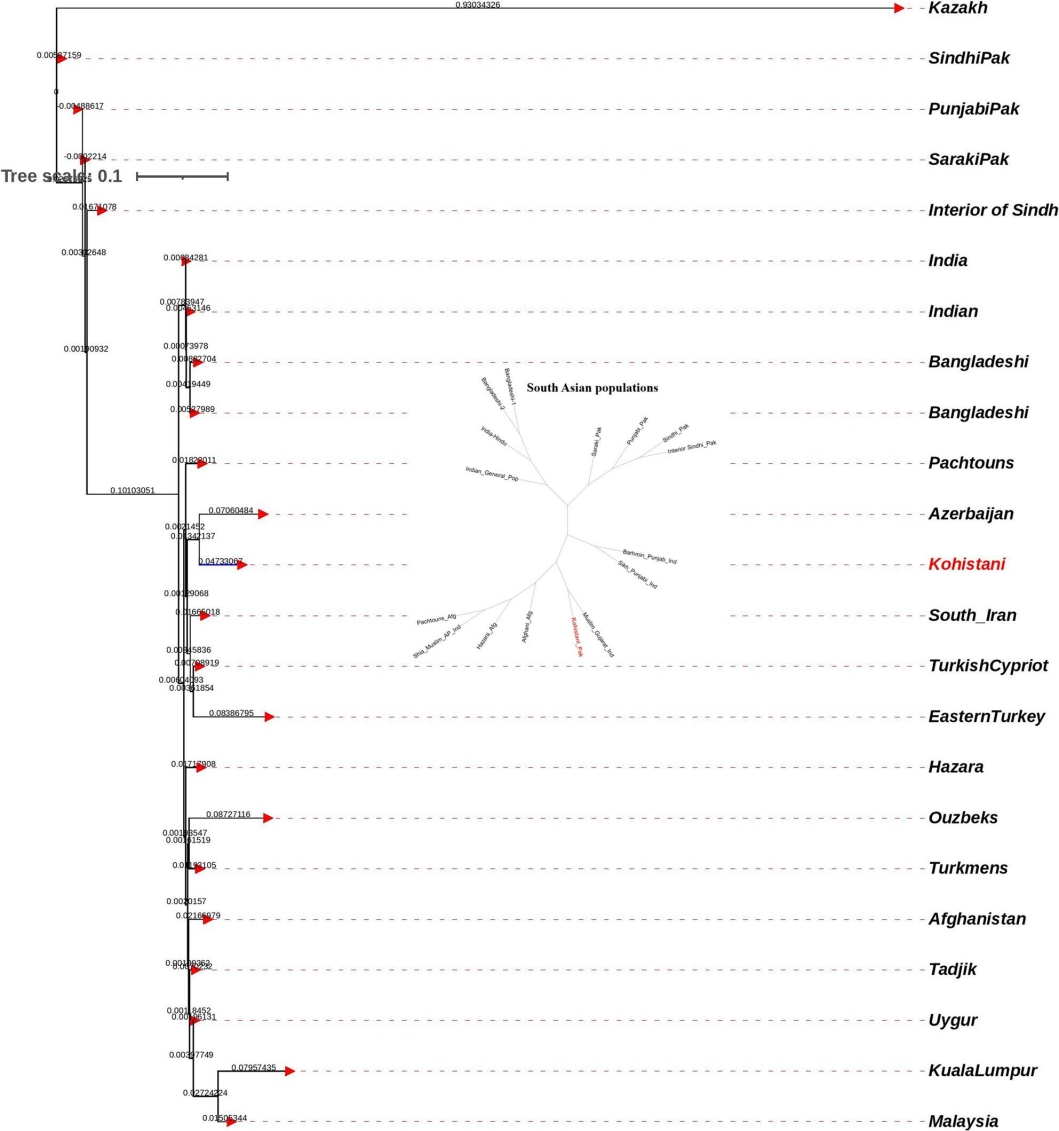
NJ Tree between Kohistani population, and 22 reginal populations based on Nei’s formula

**Fig. 8:**
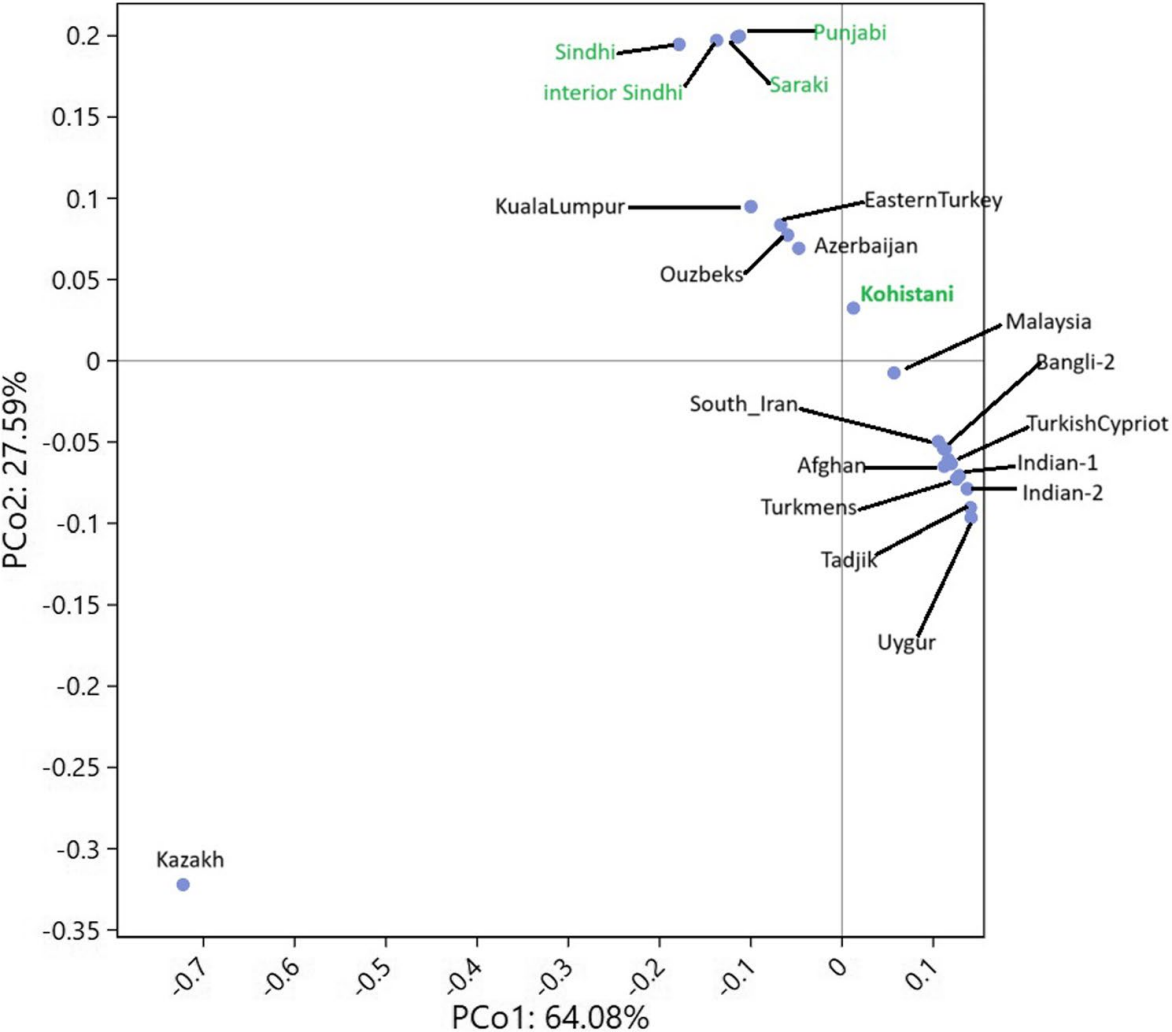
Principal component analysis (PCA) between Kohistani population, and 22 reginal populations

The multi-dimensional scaling (MDS) plot (Fig. 6) and neighbor-joining (NJ) tree (Fig. 7) consistently position Kohistani within a South Asian genetic continuum, showing strong affinity with South Asian populations like Punjabi_Pak, Saraki, Sindhi_Pak, Interior_of_Sindh, and Bangladeshi. The smallest Nei’s genetic distance is with the Indian population (0.0716), followed by the Tadjik population (0.0724). Kohistani’s proximity to Pashtuns and Bangladeshi reflects shared geographic and linguistic ties, such as their Dardic heritage and location in northern Pakistan. In contrast, greater distances from Central Asian (e.g., Uzbek: 0.111, Kazakh: 0.131) and East Asian (e.g., Uygur: 0.127, Malaysian: 0.127) populations highlight historical barriers to gene flow, such as geographic isolation and cultural differences. The Principal Coordinates Analysis (PCoA) (Fig. 8) further supports this, with PC1 (64.08%) separating South Asian populations from Central and East Asian groups, and PC2 (22.59%) showing Kohistani’s tight clustering with South Asian groups like Pashtuns and Bangladeshi.

Addressing the ethnic diversity within the South Asian cluster, the Nei’s genetic distance matrix reveals that Kohistani’s smallest genetic distance among South Asian subgroups is with Sikh_Punjabi_Ind (0.0706), followed closely by India-Hindu (0.0716) and Indian_General_Pop (0.0732). These distances are smaller than those with other subgroups, such as Bangladeshi-1 (0.0726), Bangladeshi-2 (0.0836), Punjabi_Pak (0.1701), Saraki_Pak (0.1703), Interior Sindhi_Pak (0.1805), and Sindhi_Pak (0.2083). Within Indian subgroups, Kohistani shows greater distance from Muslim_Gujarat_Ind (0.1001), Shia_Muslim_AP_Ind (0.1048), and Brahmin_Punjab_Ind (0.0971). The MDS plot (Fig. 6) visually confirms this, placing Kohistani closest to Sikh_Punjabi_Ind, India-Hindu, and Indian_General_Pop, while Punjabi_Pak, Saraki_Pak, and Sindhi_Pak are farther away. The NJ tree (Fig. 7) supports this, with Kohistani branching most closely with Sikh_Punjabi_Ind and India-Hindu, alongside Bangladeshi populations, within a tight South Asian cluster. The small branch lengths between Kohistani and Sikh_Punjabi_Ind (0.0706) suggest recent gene flow or shared ancestry, likely influenced by geographic proximity in northern Pakistan and shared cultural practices. Larger distances to groups like Sindhi_Pak (0.2083) and Muslim_Gujarat_Ind (0.1001) reflect greater genetic differentiation due to historical endogamy or isolation.

Among Bangladeshi and Bengali subgroups, Kohistani’s distance to Bangali (0.0726) is smaller than to Bangladeshi-2 (0.0836), indicating a closer affinity to the former, though both are farther than Sikh_Punjabi_Ind and India-Hindu. Among Punjabis, the distance to Sikh_Punjabi_Ind (0.0706) is much smaller than to Punjabi_Pak (0.1701) or Brahmin_Punjab_Ind (0.0971), pinpointing the Sikh Punjabi subgroup as the closest match. For Indian populations, India-Hindu (0.0716) and Indian_General_Pop (0.0732) are closer than Muslim_Gujarat_Ind (0.1001) or Shia_Muslim_AP_Ind (0.1048), highlighting Kohistani’s specific affinity with Hindu and general Indian populations over Muslim subgroups.

In conclusion, Kohistani’s closest genetic relationships within the South Asian cluster are with the Sikh_Punjabi_Ind subgroup (0.0706), followed by India-Hindu (0.0716) and Indian_General_Pop (0.0732). These findings refine the analysis by specifying that Kohistani’s strongest affinity is with Sikh Punjabis from India, likely due to shared ancestry and geographic proximity, rather than with Pakistani Punjabis (Punjabi_Pak: 0.1701) or other subgroups like Sindhi_Pak (0.2083) or Muslim_Gujarat_Ind (0.1001). Among Bangladeshi populations, Kohistani is closer to Bangladeshi-1 (0.0726) than Bangladeshi-2 (0.0836), but these are still more distant than the Sikh Punjabi and Hindu Indian subgroups. This precision accounts for ethnic diversity within South Asian populations and identifies Kohistani’s closest genetic ties within the region.

### Concluding remarks

This study provides a comprehensive analysis of the genetic structure of the Kohistani population from northern Pakistan using 21 autosomal STR loci, revealing insights into its forensic utility, genetic diversity, and population history. The high forensic efficiency of the STR panel, with a combined power of discrimination (CPD) of 0.999 999 999 999 999 999 999 999 999 999 378 and a combined power of exclusion (CPE) of 0.9999336, underscores its effectiveness for individual identification and kinship analysis in the Kohistani population. These values are comparable to those reported for other South Asian populations using the GlobalFiler™ kit, such as Pashtuns from Khyber Pakhtunkhwa (CPD: >0.999999, CPE: 0.9998–0.9999) [29] and Punjabis from Lahore (CPD: >0.999999, CPE: 0.9997–0.9999) [30]. However, the Kohistani population exhibits lower genetic diversity at certain loci, particularly TPOX (GD: 0.7389; Ho: 0.545; PD: 0.8852), compared to Pashtuns (GD: 0.73–0.92; Ho: 0.55–0.85) [29], Punjabis (GD: 0.75–0.95; Ho: 0.6–0.9) [30], and Bangladeshis (CPD: 0.99991 for four STR loci including TPOX) [32]. This reduced diversity is likely attributable to the Kohistani population’s small effective population size and strict endogamous practices, which amplify genetic drift and limit allelic variation, a pattern also observed in the Baloch population (GD: 0.74–0.90; Ho: 0.58–0.82) [31].

Initial deviations from Hardy-Weinberg equilibrium (HWE) across all loci, particularly an excess of homozygotes at TPOX (*p* < 0.05), were resolved after sequential Bonferroni correction, suggesting that these deviations stem from population substructure and inbreeding due to endogamy. Similar HWE deviations have been reported in other endogamous South Asian populations, such as certain Indian subgroups (e.g., Chandra et al., 2021 [34]), where consanguinity leads to increased homozygosity. In contrast, Pashtuns show fewer HWE deviations post-correction [29], and Punjabis often conform to HWE without correction [30], reflecting random mating in larger, less isolated populations. These findings highlight the Kohistani population’s genetic distinctiveness, shaped by its geographic isolation in the mountainous Kohistan region and cultural practices limiting gene flow.

The significant non-random associations observed in 16 pairwise locus comparisons after Bonferroni correction, particularly for loci on different chromosomes (e.g., D18S51/TPOX, D1S1656/D8S1179), are unlikely to reflect true linkage disequilibrium due to physical linkage. Instead, these patterns likely result from demographic and cultural factors, such as genetic drift, endogamy, and a small effective population size, as seen in other isolated South Asian groups like the Baloch [31]. Compared to Pashtuns, who exhibit fewer significant LD pairs (10 pre-correction) [29], and Punjabis, who show minimal LD [30], the Kohistani population’s elevated non-random associations underscore the stronger impact of isolation and inbreeding. These results align with studies of endogamous Indian populations, where increased LD is attributed to population substructure [34, 35], emphasizing the need to interpret such patterns cautiously in small, isolated populations.

Genetic distance analyses (Fst, Nei’s distances), MDS, PCoA, and STRUCTURE revealed that the Kohistani population clusters closely with South Asian populations, particularly Bangladeshi (Fst: 0.0085), Punjabi from Lahore (Fst: 0.0094), and Pashtuns (Nei’s distance: 0.0732), reflecting shared ancestry and regional gene flow. The STRUCTURE analysis at K = 4 showed a nearly exclusive green ancestry component (99.5%) in Kohistani, shared with other South Asian populations, suggesting a distinct genetic signature shaped by historical migrations and founder effects. These findings are consistent with Khan et al. (2019) [35], who reported genetic links between Kohistani and West Eurasian populations, potentially via Bronze Age migrations along the Silk Road, but our study emphasizes stronger regional continuity with South Asian groups. The Kohistani population’s greater genetic distance from Central Asian (e.g., Uzbek: 0.111) and East Asian (e.g., Uygur: 0.127) populations highlights historical barriers to gene flow, such as the rugged terrain of the Himalayas and Hindukush.

The interactivity test (Supplementary Fig. 1) further confirmed significant genetic interactions between Kohistani and South Asian populations (e.g., Bangladeshi, *p* = 0.0012; Punjabi, *p* = 0.0028), with weaker interactions with European, African, East Asian, and South American populations, reinforcing regional genetic homogeneity. These results align with the MDS and PCoA plots (Figs. 3, 6 and 8), which place Kohistani within a tight South Asian cluster, particularly close to Sikh Punjabi from India (Nei’s distance: 0.0706) and Hindu Indian populations (0.0716). Compared to published data, the Kohistani population’s genetic affinity with Sikh Punjabis and Hindu Indians is closer than with Pakistani Punjabis (0.1701) or Sindhi (0.2083) [34], likely due to shared Dardic linguistic heritage and geographic proximity in northern Pakistan.

This study contributes to forensic genetics by demonstrating the high discriminatory power of the GlobalFiler™ STR panel in the Kohistani population, supporting its use in forensic applications such as paternity testing and individual identification in this understudied region. From a population genetics perspective, the findings elucidate the Kohistani population’s unique genetic structure, shaped by endogamy and isolation, while confirming its place within the South Asian genetic continuum. The reduced diversity at loci like TPOX, combined with patterns of HWE deviations and non-random associations, mirrors findings in other endogamous groups (e.g., Baloch [31], Indian subgroups [34]), underscoring the impact of cultural practices on genetic variation. These findings are consistent with broader genomic studies of South Asia, which demonstrate a history of admixture between Ancestral South Indian (ASI) and Ancestral North Indian (ANI) populations, with Kohistani likely reflecting a predominance of ANI ancestry due to its northern geographic position [6, 7].

Future studies should incorporate mitochondrial DNA and Y-chromosomal markers to explore maternal and paternal lineages, providing a more comprehensive view of Kohistani’s population history. Expanding the dataset to include other isolated South Asian populations, such as the Kalash or Burusho, could further clarify the role of endogamy and isolation in shaping regional genetic diversity. Additionally, integrating genomic data (e.g., whole-genome sequencing) with anthropological and linguistic data will enhance our understanding of Kohistani’s historical connections to Silk Road migrations and its place within South Asia’s complex genetic landscape.

In conclusion, this study highlights the Kohistani population’s genetic distinctiveness within the South Asian context, driven by endogamy and geographic isolation, while affirming its close genetic ties to neighboring populations like Bangladeshi, Punjabi, and Pashtun. These findings enrich our understanding of South Asian genetic diversity, provide valuable forensic data, and underscore the importance of studying underrepresented populations to unravel the intricate patterns of human genetic variation.

## Supplementary Information

Supplementary Material 1: Supplementary Figure 1: Interactivity test results showing genetic relationships between the Kohistani population (KHS) and reference populations from the 1000 Genomes Project. Lower values represent stronger genetic interactions, indicating closer affinity.

Supplementary Material 2: Supplementary Table 1: The distribution of allele frequencies on 21 STRs loci in Kohistani population from KPK, Pakistan. Supplementary Table 2: Sequential Bonferroni corrections for p values from HWE exact tests on 21STRs loci in Kohistani population from KPK, Pakistan. Supplementary Table 3: Sequential Bonferroni corrections for p values from exact tests for LE on 21 STRs loci in Kohistani population from KPK Pakistan. Supplementary Table 4: Pairwise Fst genetic distances between Kohistani population from Pakistan and 26 worldwide reference populations from 1000 genome project phase 3.

## Data Availability

The full genotype dataset is not publicly shared due to the potential risk of re-identification inherent in forensic-grade genetic markers. However, anonymized data may be made available upon submission of a scientifically justified request to the authors (AA: mirzaatifadnan@gmail.com, MI: milyas@icp.edu.pk), subject to review and approval by the relevant institutional ethics and data governance authorities.
