# Supplementary figures and images for: Unveiling the genetic tapestry of Kohistan: a population genetic analysis of autosomal STRs in a South Asian population
Source: BMC Genomics. 2026 Feb 13;27:288. doi: 10.1186/s12864-026-12641-x (PMC13005355; doi:10.1186/s12864-026-12641-x)

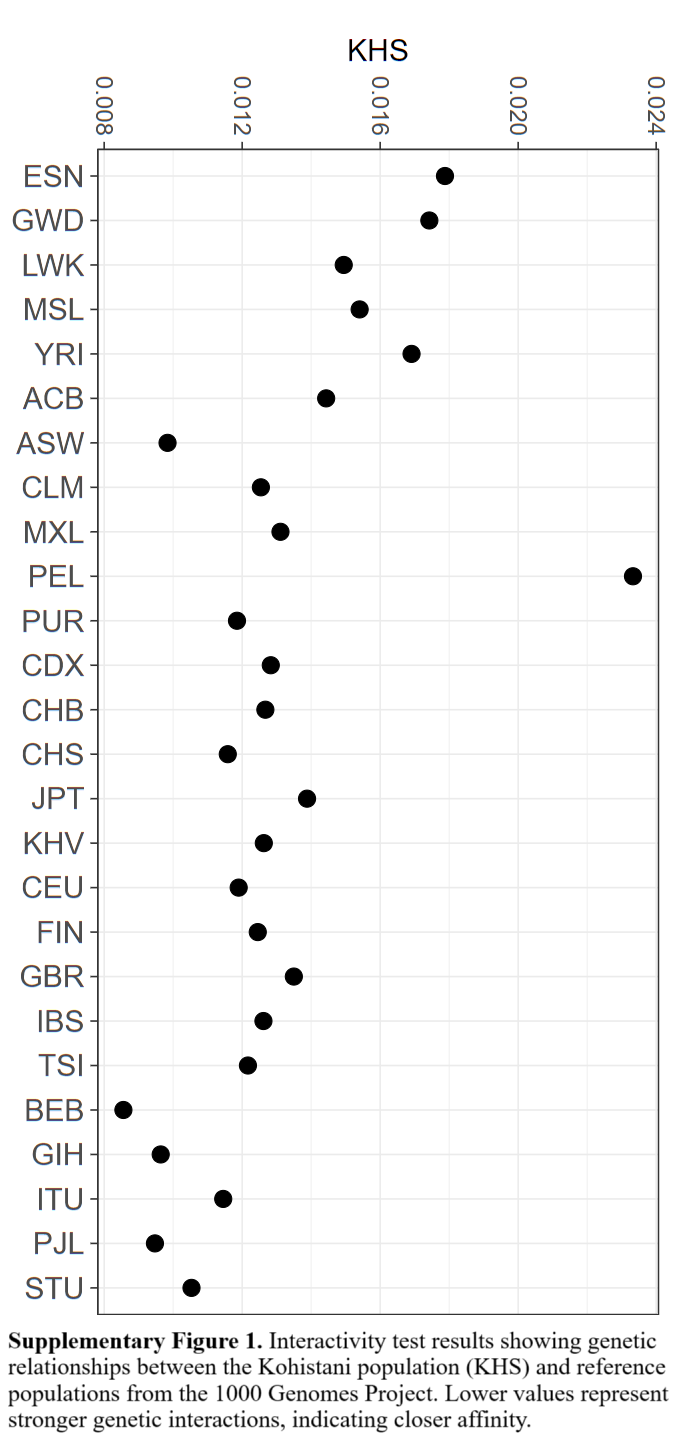

Supplement: Supplementary file 1 — Supplementary Material 1: Supplementary Figure 1: Interactivity test results showing genetic relationships between the Kohistani population (KHS) and reference populations from the 1000 Genomes Project. Lower values represent stronger genetic interactions, indicating closer affinity. [file 12864_2026_12641_MOESM1_ESM.png]
